# Inflammatory pseudotumors of the kidney and the lung presenting as immunoglobulin G4-related disease: a case report

**DOI:** 10.1186/1752-1947-5-480

**Published:** 2011-09-25

**Authors:** Genya Nishikawa, Kogenta Nakamura, Yoshiaki Yamada, Takahiko Yoshizawa, Yoshiharu Kato, Remi Katsuda, Kenji Zennami, Motoi Tobiume, Shigeyuki Aoki, Tomohiro Taki, Nobuaki Honda

**Affiliations:** 1 Department of Urology, Aichi Medical University School of Medicine, Nagakute, Aichi 480-1195, Japan

## Abstract

**Introduction:**

It has been reported that immunoglobulin G4-related systemic disease can spread to nearly every organ, and often presents as an inflammatory mass or masses at those sites. In the kidney, this disease is often diagnosed after a radical or partial nephrectomy following the discovery of an inflammatory mass which is often suspected to be a malignant tumor. Here, we present a rare case of inflammatory pseudotumors of the kidney and the lung presenting as immunoglobulin G4-related disease, which were diagnosed by computed tomography-guided biopsies.

**Case presentation:**

A 54-year-old Japanese man was referred to our hospital with suspected bilateral renal cancer, multiple lung metastases and autoimmune pancreatitis. His serum immunoglobulin G4 level was high. We used computed tomography-guided biopsies and histopathological examinations of the biopsied specimens to diagnose the tumors as immunoglobulin G4-related bilateral renal and lung inflammatory pseudotumors. Our patient was treated with oral prednisolone, and after one month of treatment, contrast-enhanced computed tomography demonstrated a general improvement, as noted by a reduction in size of the masses.

**Conclusion:**

Renal masses that are formed due to immunoglobulin G4-related disease require comprehensive diagnosis to prevent unnecessary surgical resections from being performed. Further consideration should be paid to immunoglobulin G4-related diseases in the future.

## Introduction

Patients with autoimmune pancreatitis often exhibit high serum immunoglobulin G4 (IgG4) levels, and/or marked infiltration of IgG4-positive plasma cells, both of which are hallmarks of IgG4-related systemic disease. In addition to the pancreas, IgG4-related mass-forming lesions have also been described in other organs. In the kidney, IgG4-related disease can also present as an inflammatory mass, and is often diagnosed after a radical or partial nephrectomy due to suspected malignancy. Here, we present a case report of a patient with IgG4-related bilateral renal and lung inflammatory pseudotumors. We also describe the diagnostic process and treatment course.

## Case presentation

**Figure 1:**
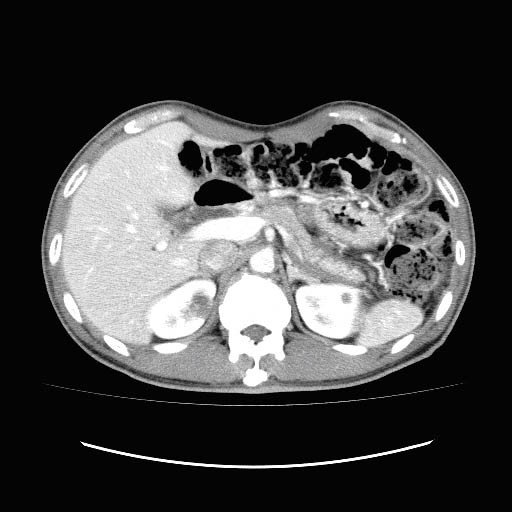
**CT showed poorly enhanced mass lesions (left, 10 × 10 mm; right, 18 × 14 mm) in the upper part of both kidneys**.

**Figure 2:**
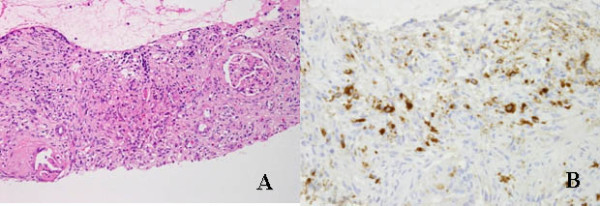
**Right renal mass**. (A) Fibrous tissue involving glomeruli, with infiltration of lymphocytes and plasma cells (hematoxylin and eosin staining, low power); (B) IgG4-positive plasma cells (44 cells/HPF, IgG4 immunostaining).

A 54-year-old Japanese man presented to a Department of Internal Medicine with chief complaints of dry mouth and weight loss that he had been experiencing for two months. He had type 1 diabetes mellitus, which was being treated with insulin. Computed tomography (CT) scans showed the presence of bilateral renal and pulmonary masses, and swelling of his pancreatic parenchyma. He was referred to our hospital with suspected bilateral renal cancer, multiple lung metastases and autoimmune pancreatitis. He had bronchial asthma, but no habitual contributory factors. Blood tests revealed high IgG (1775 mg/dL) and IgG4 levels (351 mg/dL). Lactate dehydrogenase and C-reactive protein levels, which are prognostic factors for renal cancer, were normal (162U/L and 0. mg/dL, respectively). No other abnormal values were noted, including urine analysis results. Contrast-enhanced CT scans showed multiple nodular opacities of various sizes with irregular margins in both lung fields. In his abdomen, there were slight poorly enhanced mass lesions (left, 10 × 10 mm; right, 18 × 14 mm) in the upper pole of both kidneys (Figure 1). Magnetic resonance imaging showed a mass in each kidney with a low signal intensity on the T1- and T2-weighted images, and poorly enhanced areas inside each mass. Magnetic resonance cholangiopancreatography and endoscopic retrograde cholangiopancreatography were performed, and our patient was diagnosed with autoimmune pancreatitis. The bilateral renal and pulmonary masses were suspected of being multiple lung metastases stemming from bilateral renal cancer, but inflammatory pseudotumors associated with autoimmune pancreatitis could not be ruled out. Based on these findings, a CT-guided biopsy was performed on the right pulmonary mass and right renal mass. Histopathological examination of the biopsy specimens showed extensive fibrous tissue around the glomeruli in his kidney and alveoli in his lung. Infiltration of lymphocytes and plasma cells was also observed. A malignant tumor was considered unlikely because no atypical cells were observed. However, immunohistochemical staining revealed the presence of IgG- and IgG4-positive plasma cells; the number of IgG4-positive plasma cells was 44 cells per high power field (HPF) as shown in Figure 2. Based on these findings, we diagnosed the tumor as IgG4-related bilateral renal and multiple pulmonary masses.

**Figure 3:**
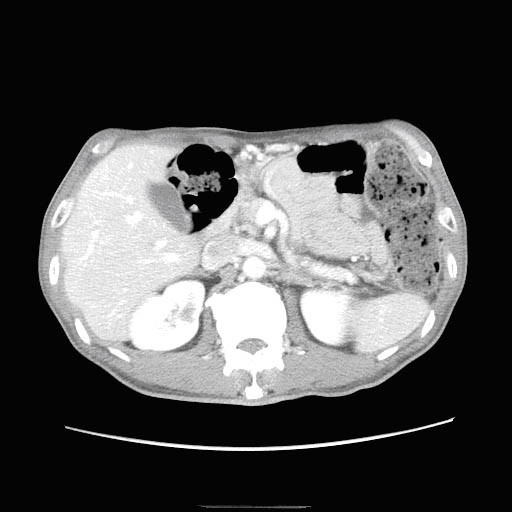
**CT showed reduction of the right renal mass, and disappearance of the left renal mass**.

Following diagnosis, treatment with oral prednisolone (40 mg/day) was initiated. A CT scan performed on the ninth day of treatment showed a reduction in the size of the masses, so the dose was decreased to 30 mg. Subsequent CT scans showed a further reduction in the size of the masses so treatment with prednisolone was tapered. After one month of treatment, contrast-enhanced CT revealed new, small pulmonary lesions, but also demonstrated a general improvement, with a reduction in size of the right renal mass, and disappearance of the left renal mass (Figure 3). Serum IgG levels decreased to values within the normal range after initiation of treatment with prednisolone. Our patient continued receiving 10 mg prednisolone per day to prevent a recurrence, which to date, has not occurred.

## Discussion

In 2001, Hamano *et al*. first reported that patients with autoimmune pancreatitis have high serum IgG4 levels and infiltration of IgG4-positive plasma cells [1]. Subsequently, it was reported that several patients had developed mass-forming lesions in organs other than the pancreas. Thus, a novel disease entity was proposed and termed, 'IgG4-related systemic disease'. IgG is an immunoglobulin consisting of four subclasses (IgG1, IgG2, IgG3, and IgG4). IgG4 is the rarest IgG subclass in the serum, and accounts for 6% or less of total IgG. Zen *et al*. reported that Th2 cells are predominant in lesions of IgG4-related disease that are associated with an infiltration of numerous CD4- and CD25-positive regulatory T cells (Tregs). Furthermore, the authors found that interleukin-10 and tumor growth factor-β secreted from Tregs are involved in the proliferation of IgG4-positive plasma cells, excess IgG4 secretion, fibrosis and tumor formation [2].

IgG4-related systemic disease occurs more frequently in middle-aged and older men, with a male to female ratio of 2:1. The most common sites of occurrence are the pancreas and lung, but it can also occur in other tissues and organs including the common bile duct, salivary glands, kidney, prostate and retroperitoneum. In the kidney, IgG4-related disease sometimes manifests itself as tubulointerstitial nephritis, in which tumor formation is not a feature [3]. A definitive diagnosis of these diseases requires histopathological evidence of inflammatory cell infiltration, particularly IgG4-positive plasma cells. Lynn *et al*. proposed that IgG4-related disease should be definitively diagnosed based on the presence of at least 30 cells/HPF of IgG4-positive plasma cells in a lesion [4]. IgG4-related diseases also involve the formation of masses that are characterized by marked fibrosis and obliterating phlebitis.

It is important to differentiate these diseases from malignant tumors. To this end, for the diagnosis of autoimmune pancreatitis, the clinical diagnostic criteria were revised in 2006 to include high IgG4 levels and a mass-forming lesion outside the pancreas [5]. There are no established guidelines regarding the treatment of IgG4-related systemic diseases, so we based our treatment on the Japanese consensus guidelines for the management of autoimmune pancreatitis, as recommended by the Japan Pancreas Society. Treatment was initiated with 0.6 kg/mg/day prednisolone, and the dose was tapered to 5 mg every one to two weeks according to the clinical symptoms and results upon examination or imaging. To prevent recurrence, maintenance doses of 5 mg were required for three years [6].

The renal and pulmonary masses in our patient were suspected to be bilateral renal cancer and multiple lung metastases. However, inflammatory pseudotumors could not be ruled out, because our patient also had autoimmune pancreatitis and high serum IgG and IgG4 levels. Therefore, a CT-guided biopsy and pathological examination were performed, leading to the diagnosis of IgG4-related disease. There are some reports of patients who presented with a renal mass, and thus underwent a nephrectomy as the mass was mistaken for a malignant tumor. Many of these patients were subsequently diagnosed with IgG4-related systemic disease by pathological examination [7,8]. Since those patients did not have any clear symptoms aside from a renal mass, it seemed impossible to suspect IgG4-related disease based solely on imaging results. One reason for this may be that neither a definitive description of IgG4-related disease nor diagnostic criteria have been established. However, there are some types of prostatitis, idiopathic retroperitoneal fibrosis and renal masses that have been characterized as IgG4-related systemic diseases, and which are familiar to urologists.

## Conclusion

We have presented a case of IgG4-related systemic disease characterized by masses in the kidney and lung, and diagnosed by CT-guided biopsy. Renal masses in particular require comprehensive diagnosis so that unnecessary surgical resection can be avoided. Further emphasis should be placed on the study of IgG4-related diseases in the future.

## Consent

Written informed consent was obtained from the patient for publication of this case report and any accompanying images. A copy of the written consent is available for review by the Editor-in-Chief of this journal.

## Competing interests

The authors declare that they have no competing interests.

## Authors' contributions

GN drafted the report. TY, YK, RK, KZ, MT, SA and TT cared for the patient. YY and NH reviewed the report. KN drafted the report and cared for the patient. All authors read and approved the final version of the manuscript.
